# 

**Published:** 1894-09

**Authors:** 


*The following donations have been received since the publication of the list in June :*

*August 31st, 1894.*

| | |
|---|---|
| R. E. Burges, M.D. (1) | 8 volumes. |
| G. G. Corbould (2) | 3 volumes. |
| W. Duncan (3) | 65 volumes. |
| W. H. Harsant (4) | 1 volume. |
| J. Greig Smith (5) | 2 volumes. |

### THIRTEENTH LIST OF BOOKS.

The figures in brackets refer to the figures after the names of the donors, and show by whom the volumes were presented. The books to which no such figures are attached have either been bought from the Library Fund or received through the *Journal.*

| | | | |
|---|---|---|---|
| **Aitken, W.** | *The Science and Practice of Medicine.* 2 vols. | (3) 4th Ed. | 1866 |
| **Anderson, T. M'Call** | *A Treatise on Diseases of the Skin* | 2nd Ed. | 1894 |
| **Ball, J. B.** | *Diseases of the Nose and Pharynx* | 2nd Ed. | 1894 |
| **Begbie, J.** | *Contributions to Practical Medicine* | (3) | 1862 |
| ,, **J. W.** | *Address in Medicine* | (3) | 1875 |
| **Bennett, J. H.** | *Text-book of Physiology.* Parts 1—3 | (3) | 1872 |
| **Bentley, A. J. M.** | *Béri-Béri* | | 1893 |
| **Bidlake, J. P.** | *Text-book of Elementary Chemistry* | (3) 8th Ed. | [N.D.] |
| **Bigg, H.** | *Artificial Limbs* | (3) | 1885 |
| **Bignami, E. Marchiafava and A.** | *On Summer-Autumn Malarial Fevers,* (Tr. by J. H. Thompson) | | 1894 |
| **Blake, E. T.** | *Myxœdema, Cretinism and the Goitres* | | 1894 |
| **Braidwood, P. M.** | *The Mother's Help and Guide* | | 1894 |
| **Browne, R.** | *Neurasthenia and its Treatment by Hypodermic Transfusions* | | 1894 |
| **Brown-Séquard, C.E.** | *Physiology and Pathology of the Central Nervous System* | (3) | 1860 |
| **Buck, A. H.** (Ed.) | *Reference Handbook of the Medical Sciences.* | (1) Vols. i—viii, 1888-90; ix, | 1893 |
| **Buckmaster, J. C.** | *The Elements of Inorganic Chemistry.* 8th Ed. (Ed. by G. Jarmain) | (3) | 1870 |
| **Bury, J. S.** | *Clinical Medicine* | | 1894 |
| **Cappie, J.** | *Essays in Medical Science* | (3) | 1859 |
| **Carpenter, W. B.** | *The Microscope* | (3) 4th Ed. | 1868 |
| **Carter, W.** | *Midwives' Education and Registration* | | 1894 |
| **Churchill, F.** | *Diseases of Women* | (3) 4th Ed. | 1857 |
| ,, | *Theory and Practice of Midwifery* | (3) 6th Ed. | 1872 |

**Cooke, T.** ... ... *Tablets of Anatomy and Physiologv—Anatomy* ... (3) 1875

**Day, W. H.** ... *Headaches* ... ... ... ... ... ... ... (3) 2nd Ed. 1878

*Dictionary of Mineral Waters, Climatic Health Resorts, &c.* ... ... ... ... ... 1894

**Druitt, R.** ... ... *The Surgeon's Vade-Mecum* ... ... ... (3) 10th Ed. 1870

**Ferguson, F. W. N. Haultain and J. H.** *Handbook of Obstetric Nursing.* 2nd Ed. 1894

**Fergusson, Sir W.** *A System of Practical Surgery* ... ... (3) 5th Ed. 1870

**Flint, A.** ... ... *Principles and Practice of Medicine* ... (3) 4th Ed. 1873

**Font. M.** ... ... *De la Filariosis* ... ... ... ... ... ... .. ... ... 1894

**Fox, C. B.** ... ... *Ozone and Antozone* ... ... ... ... ... ... ... (3) 1873

**Gleason, E. Jackson and E. B.** *Essentials of Diseases of the Eye, Nose, and Throat* ... ... ... ... ... ... 2nd Ed. 1894

**Hart, E.** ... ... *Essays on State Medicine.* 2 vols. ... ... ... ... 1894

,, ... ... *On the Use of Opium in India* ... ... ... ... ... ... 1894

**Hartmann, A.** ... *Deafmutism.* (Tr. by J. P. Cassells) ... ... ... (4) 1881

**Haultain and J. H. Ferguson, F. W. N.** *Handbook of Obstetric Nursing.* 2nd Ed. 1894

**Holmes, T.** (Ed.)... *A System of Surgery.* 5 vols. ... (3) 2nd Ed. 1869-71

**Hooper, R.** ... ... *Physician's Vade-Mecum.* 8th Ed. (Ed. by W. A. Guy and J. Harley ).... ... ... ... ... ... (3) 1869

**Hutchinson, J.** ... *A Descriptive Catalogue of the Clinical Museum.* Part I. [1894]

**Jackson and E. B. Gleason, E.** *Essentials of Diseases of the Eye, Nose, and Throat* ... ... ... ... 2nd Ed. 1894

**Jones and E. H. Sieveking, C. H.** *Manual of Pathological Anatomy.* (Ed. by J. F. Payne)... ... (3) 2nd Ed. 1875

**Keith, S. and G. E.** *Text-book of Abdominal Surgery* ... ... ... ... ... 1894

**Kerr, N.** ... ... *Inebriety or Narcomania* ... ... ... ... ... 3rd Ed. 1894

**Leishman, W.** ... *A System of Midwifery* ... ... ... ... (3) 2nd Ed. 1876

**Mackay, G.**... ... *On Blinding of the Retina by Direct Sunlight* ... ... 1894

**Mannaberg, J.** ... *The Malarial Parasites.* (Tr. by R. W. Felkin) ... 1894

**Marchiafava and A. Bignami, E.** *On Summer-Autumn Malarial Fevers.* (Tr. by J. H. Thompson) ... ... 1894

**Mather, G. R.** ... *Two Great Scotsmen—William and John Hunter*... ... 1893

**Mercier, C.** ... ... *Lunacy Law for Medical Men* ... .. ... ... ... 1894

**Moure, E. J.** ... *Contribution a l'étude de l'ulcère perforant de la cloison du nez* ... ... ... ... ... ... ... ... ... ... ... 1894

**Murchison, C.** ... *Diseases of the Liver, &c.* ... ... ... ... ... (3) 1868

**Mygind, H.**... ... *Deaf-Mutism* ... ... ... ... ... ... ... ... ... ... 1894

**Oliver, G.** ... ... *Bedside Urine-Testing* ... ... ... ... (3) 3rd Ed. 1885

**Parker, C. A.** ... *Post-Nasal Growths* ... ... ... ... ... ... ... 1894

**Pavy, F. W.** ... *The Physiology of the Carbohydrates* ... ... ... ... 1894

*Pharmacopœia of the Hospital for Diseases of the Throat, The* ... 5th Ed. 1894

**Pinard, A.** ... ... *De l'Agrandissement momentané du Bassin* ... ... ... 1894

**Pirrie, W.** ... ... *The Principles and Practice of Surgery* ... (3) 3rd Ed. 1873

**Quain, J.** ... ... *Elements of Anatomy.* 10th Ed. (Ed. by E. A. Schäfer and G. D. Thane). Vol. III. Part III. ... ... 1894

**Ramsbotham, F. H.** *Obstetric Medicine and Surgery* ... ... (3) 4th Ed. 1856

**Reynolds, J. R.** (Ed.) *A System of Medicine.* 5 vols. ... ... ... ...(3) 1866-76

**Roberts, A.**... ... *The Harrogate Mineral Waters* ... ... ... ... ... 1894

**Roose, R.** ... ... *Gout* ... ... ... ... ... ... ... ... ... ... 7th Ed. 1894

**Rutherford, W.** ... *Outlines of Practical Histology* ... ... ... ... ... (3) 1875

**Sieveking, C. H. Jones and E. H.** *Manual of Pathological Anatomy.* (Ed. by J. F. Payne) (3) 2nd Ed. 1875

**Smith, N.** ... ... *Spinal Caries* ... ... ... ... ... ... ... ... ... ... ... 1894

**Syme, J.** ... ... *The Principles of Surgery* ... ... ... (3) 4th Ed. 1856

,, ... ... *Observations in Clinical Surgery* ... ... ... ... (3) 1861

,, ... ... *Excision of the Scapula* ... ... ... ... ... ... ... (3) 1864

**Taylor, A. S.** ... *On Poisons* ... ... ... ... ... ... ... (3) 2nd Ed. 1859

**Thomas, J. D.** ... *Hydatid Disease.* Vol. II. (Ed. by A. A. Lendon) 1894

**Thorne, W. B.** ... *The Treatment of Chronic Diseases of the Heart by Baths and Exercises* ... ... ... ... ... ... ... ... 1894

**Tuke, J. B.** ... ... *The Insanity of Over-exertion of the Brain* ... ... ... [1894]

**Urquhart, J. W.** ... *Electro-Typing* ... ... ... ... ... ... ... ... (3) 1881

**Watson, T.** ... *Lectures on the Principles and Practice of Physic.* 2 vols. (3) 4th Ed. 1857

**Winslow, F.** ... *On Obscure Diseases of the Brain* ... ... (3) 2nd Ed. 1861

### TRANSACTIONS, REPORTS, JOURNALS, &c.

American Journal of the Medical Sciences, The ... ... Vol. CVII. 1894

Archives de Neurologie ... ... ... ... ... ... ... ... Tome XXVII. 1894

Birmingham Medical Review, The ... ... ... ... ... Vol. XXXV. 1894

Bristol Health Report for 1893 ... ... ... ... ... ... ... ... ... 1894

British Medical Journal ... ... ... ... ... ... (3) 1885-92; Vol. I. for 1894

Clinical Journal, The ... ... ... ... ... ... ... ... ... Vol. III. 1894

Dublin Journal of Medical Science, The ... ... ... ... Vol. XCVII. 1894

Edinburgh Medical Journal ... ... ... ... ... ... ... ...Vol. XXXIX. 1894

Hospital, The ... ... ... ... ... ... ... ... ... ... ... ...Vol. XV. 1894

Hospitals—

Edinburgh Hospital Reports ... ... ... ... ... ... Vol. II. 1894

Guy's Hospital Reports ... ... ... ... ... ... ... ... Vol. L. 1894

Johns Hopkins Hospital Reports, The ... Vol. IV., Nos. 4-5 1894

Middlesex Hospital—Registrars' Reports for 1892... ... ... ... 1894

Journal of Microscopy and Natural Science, The ... ... ... Vol. III. 1893

Lancet, The ... ... ... ... ... ... ... ... ... ... ... ...Vol. I for 1894

Library, The ... ... ... ... ... ... ... ... ... ... ... ... Vol. V. 1893

Medical Directory, The ... ... ... ... ... ... ... ... ... (3) 1886, 1888

Medical Magazine, The ... ... ... ... ... ... ... ... ... Vol. II. 1894

Medical News, The ... ... ... ... ... ... ... ... ... ... Vol. LXIV. 1894

Medical Press and Circular, The ... ... ... ... ... ... Vol. CVIII. 1894

Medical Record ... ... ... ... ... ... ... ... ... ... ... Vol. XLV. 1894

New York Medical Journal, The ... ... ... ... ... ... Vol. LIX. 1894

Practitioner, The (2) Vols. XL.—XLII., 1888-89; (5) XLIV.—XLV,, 1890; XLVI.—XLVII., 1891; L—LII., 1893-94

Progrès Médical, Le ... ... ... ... ... ... ... ... ... ... Tome XIX. 1894

Retrospect of Medicine, Braithwaite's ... ... ... ... Vol. CIX. 1894

Union Médicale, L' ... ... ... ... ... ... ... ... ... ... Tome LVII. 1894

Year-Book of the Scientific and Learned Societies ... ... ... ... ... 1894

---

At page 77 line two from bottom, for 1849 *read* 1894.

From THE CHURCH DEFENCE INSTITUTION, London:
*The National Church.* April—September, 1894.

From COMPAÑIA SUD-AMERICANA DE BILLETES DE BANCO, Buenos Aires:
*Revista de la Sociedad Médica Argentina.* Vol. III., Nos. 14, 15. 1894.

From DAVIES & SON, Gloucester:
*Barnwood House Hospital for the Insane.* Thirty-fourth Annual Report, for 1893. 1894.

From T. DOBB & CO., Liverpool;
*Midwives' Education and Registration.* By WILLIAM CARTER. 1894.

From OCTAVE DOIN, Paris:

*Contribution à l'étude de l'Ulcère perforant de la Cloison du Nez.* E. J. MOURE. 1894.

From O. DOIN ET FERET ET FILS, Paris—Bordeaux:
I.—*De l'Empyème du Sinus sphénoïdal.* II.—*Un Cas d'Angiokératome de la Corde vocale droite.* Dr. E. J. MOURE. 1894.
*De l'Extraction des Osselets dans l'Otorrhée.* Dr. E. J. MOURE. 1894.

From E. & J. GIBBONS, Liverpool:
*The Proposed Formation of an Inferior Order of Midwifery Practitioners.* By ROBERT REID RENTOUL. [1894.]

From CHARLES GRIFFIN & COMPANY, LIMITED, London:
*Clinical Medicine.* By JUDSON S. BURY, M.D. 1894.
*A Treatise on Diseases of the Skin.* By T. M'CALL ANDERSON, M.D. Second Edition. 1894.

From H. HELBING, London:
*Helbing's Pharmacological Record.* Nos. XXVI.—XXIX., Feb.—July.
*The Properties and Advantages of Symphorol.* By H. HELBING and Dr. F. W. PASSMORE. April, 1894.

From HENRICH Y COMPAÑIA, Barcelona:
*De la Filariosis.* Por el Dr. M. FONT Y TORNÉ. 1894.

From ILLUSTRATED LONDON NEWS OFFICE, London:
*The English Illustrated Magazine.* July, 1894.

From THE JOHNS HOPKINS PRESS, Baltimore:
*The Johns Hopkins Hospital Reports.* Vol. IV., Nos. 4—5. *Report in Neurology*, II. 1894.

From Dr. A. B. JUDSON, New York.
*The Importance of Early Attention to the Disability Caused by Infantile Paralysis.* By A. B. JUDSON, M.D. 1893. [Reprint.]

From H. K. LEWIS, London:
*Gout.* By ROBSON ROOSE, M.D. Seventh Edition. 1894.
*Post-Nasal Growths.* By CHARLES A. PARKER. 1894.
*Inebriety or Narcomania.* By NORMAN KERR, M.D. Third Edition. 1894.

From THE LONDON AND UNIVERSAL BANK, LIMITED, London:
*Everybody's Pocket Cyclopædia.* [N.D.]

From OLIVER AND BOYD, Edinburgh:
*The Insanity of Over-exertion of the Brain.* By J. BATTY TUKE, M.D. [1894.]

From KEGAN PAUL, TRENCH, TRÜBNER & CO., LTD., London:
*Defective Articulation resulting from Cleft Palate.* By WILLIAM VAN PRAAGH. Second Edition. 1894.

From YOUNG J. PENTLAND, Edinburgh:
*Text-Book of Abdominal Surgery.* By SKENE KEITH, assisted by GEORGE E. KEITH, M.B. 1894.
*Edinburgh Hospital Reports.* Volume Second. 1894.
*Bèri-Bèri.* By ARTHUR J. M. BENTLEY, M.D. 1893.
*Treatment of Wounds, Abscesses, and Ulcers.* By W. WATSON CHEYNE, M.B. 1894.

From F. J. REBMAN, London:
*Deaf-Mutism.* By HOLGER MYGIND, M.D. 1894.

